# PReS-FINAL-2286: Mastitis in an adolescent patient with juvenile systemic lupus erythematous

**DOI:** 10.1186/1546-0096-11-S2-P276

**Published:** 2013-12-05

**Authors:** BE Bica, LMR Saldarriaga, ACP Rocha

**Affiliations:** 1 Rheumatology, Universidade Federal do Rio de Janeiro, Rio de Janeiro, Brazil; 2 Mastology, Universidade Federal do Rio de Janeiro, Rio de Janeiro, Brazil

## Introduction

Mastitis is an inflammatory disease of the breast, acute or chronic that occurs primarily in young women and frequently lactating. In 2-3% it may occur in patients with systemic lupus erythematous (SLE). The authors describe in an adolescent with JSLE this rare disease called lupus mastitis, which is a subset of lupus panniculitis limited to the breast.

## Objectives

To describe a young patient with lupus mastitis.

## Methods: Case report

A 16 year-old girl presented with a painful left breast mass associated with localized skin erythema. Her medical history was significant for diffuse proliferative lupus nephritis that was diagnosed in 2002. After 2 years of immunosuppression therapy, the patient had stable renal function for more than 8 years. In January 2013, she presented with a painful nodule in the left breast. At the time of presentation, she had no clinical complaints but the serology findings were positive. There was no history of trauma to the breast, oral contraceptive use and negative sexual history. She was being treated with low dose prednisolone and azathioprine. The mass was warm and tender and no lymphadenopathy was present. The contralateral breast was normal. The laboratory tests showed normal blood count and negative inflammatory tests, ANA: 1/640 homogeneous pattern, anti-DNA: 1/80. Breast ultrasound revealed multiple areas with thick liquid collections in the subareolar region of the left breast. MRI confirmed the multiple cystic areas with subcutaneous thickening. An incisional biopsy revealed chronic inflammation, compromising lobes and stromal, compatible with inflammatory mastitis. Cultures were negative. The patient was treated with surgical drainage of collections associated to pulse therapy with methylprednisolone and mycophenolate mofetil was introduced featuring full resolution of the inflammatory process.

## Results

Mastitis is a rare manifestation observed in SLE. The pathophysiology of lupus mastitis is unknown. One thought is that the panniculitis is an extension of the inflammatory process that involves the overlying skin as epidermal changes, atrophy and ulceration may be present. This inflammatory condition can simulate a neoplasm or a breast abscess. This disorder was a challenge to diagnose for all involved physicians.

## Conclusion

Lupus mastitis is a rare chronic inflammatory reaction of the subcutaneous fat that may occur in 2-3% of patients with systemic lupus erythematous usually between the ages of 20 and 50 years, and its occurrence is two times greater in women than in men. The occurrence in a young patient is very rare e should be promptly treated with immunosuppression. The clinical course of lupus mastitis is often chronic with flares and remissions. Surgical excision alone may not cure the patient if there is inadequate immunosuppression.

## Disclosure of interest

None declared.

